# Immunoreactivity of LMO7 and other molecular markers as potential prognostic factors in oropharyngeal squamous cell carcinoma

**DOI:** 10.1186/s12903-024-04510-4

**Published:** 2024-06-25

**Authors:** Pernilla Israelsson, Husam Oda, Charlotte Öfverman, Kristina Stefansson, David Lindquist

**Affiliations:** 1 Department of Diagnostics and Intervention, Oncology, Umeå University, Umeå, 90185 Sweden; 2 Department of Medical Biosciences, Pathology, Umeå University, Umeå, 90185 Sweden; 3 Department of Medical Biosciences, Clinical Chemistry, Umeå University, Umeå, 90185 Sweden; 4 Department of Clinical Sciences, Professional Development, Umeå University, Umeå, 90185 Sweden

**Keywords:** Oropharyngeal squamous cell carcinoma, OPSCC, Head and neck cancer, Tonsillar cancer, LMO7, LRIG1, HPV, Ki67, CD44, p53

## Abstract

**Background:**

Despite the better prognosis associated with human papillomavirus (HPV)-positive oropharyngeal squamous cell carcinoma (OPSCC), some patients experience relapse and succumb to the disease; thus, there is a need for biomarkers identifying these patients for intensified treatment. Leucine-rich repeats and immunoglobulin-like domain (LRIG) protein 1 is a negative regulator of receptor tyrosine kinase signaling and a positive prognostic factor in OPSCC. Studies indicate that LRIG1 interacts with the LIM domain 7 protein (LMO7), a stabilizer of adherence junctions. Its role in OPSCC has not been studied before.

**Methods:**

A total of 145 patients diagnosed with OPSCC were enrolled. Immunohistochemical LMO7 expression and staining intensity were evaluated in the tumors and correlated with known clinical and pathological prognostic factors, such as HPV status and LRIG1, CD44, Ki67, and p53 expression.

**Results:**

Our results show that high LMO7 expression is associated with significantly longer overall survival (OS) (*p* = 0.044). LMO7 was a positive prognostic factor for OS in univariate analysis (HR 0.515, 95% CI: 0.267–0.994, *p* = 0.048) but not in multivariate analysis. The LMO7 expression correlated with LRIG1 expression (*p* = 0.048), consistent with previous findings. Interestingly, strong LRIG1 staining intensity was an independent negative prognostic factor in the HPV-driven group of tumors (HR 2.847, 95% Cl: 1.036–7.825, *p* = 0.043).

**Conclusions:**

We show for the first time that high LMO7 expression is a positive prognostic factor in OPSCC, and we propose that LMO7 should be further explored as a biomarker. In contrast to previous reports, LRIG1 expression was shown to be an independent negative prognostic factor in HPV-driven OPSCC.

## Introduction

Oropharyngeal squamous cell carcinoma (OPSCC), most often originating from the tonsils or base of the tongue, is a relatively rare group of diseases, accounting for approximately 0.5% of all new cancer cases each year [1]. However, human papillomavirus (HPV)-associated OPSCC has one of the most rapidly increasing incidence rates of all cancers [2]. In the most recent TNM staging system, stratification by HPV status is recommended, in accordance with findings of distinct molecular profiles, tumor characteristics, and outcomes [2, 3]. The prognosis is substantially better for patients with HPV-driven tumors, even though approximately 15% of them have recurrent disease [4]. HPV status does not affect current treatment recommendations. Still, considering the younger age and better prognosis associated with HPV-driven tumors, means are taken to de-escalate treatment to increase the quality of life for this group of patients [5]. Identifying HPV-positive patients with a more aggressive disease remains a challenge, and further prognostic and predictive markers are thus needed.

The human LRIG (leucine-rich repeats and immunoglobulin-like domains) family of integral transmembrane proteins comprises LRIG1, LRIG2, and LRIG3 [6]. LRIG proteins are ubiquitously expressed in human tissues and even though their role in tumorigenesis is not fully understood, LRIG proteins have a prognostic impact in various tumor diseases (reviewed in [7, 8]). LRIG1 functions as a tumor suppressant and has proven to be a negative regulator of receptor tyrosine kinase signaling [9], as well as a promotor of bone morphogenic protein (BMP) signaling [10]. LRIG1 expression has been shown to correlate with HPV status in OPSCC [11] as well as in other cancers [12, 13]. In HPV-driven OPSCC, LRIG1 expression was also proven to be an independent prognostic marker associated with longer survival [11].

Studies have shown that there is an interaction between LRIG1 and the LIM domain 7 protein (LMO7) [14]. LMO7 has a stabilizing role in adherence junctions as an afadin- and a-actinin-binding protein [15], is involved in cell migration [16], and acts as a transcription factor of muscle-related genes [17]. It has limited expression in normal tissues but is expressed in different cancers and is suggested to have an important function in cancer development and metastasis [16, 18, 19]. Its expression as a prognostic marker has been studied in several tumor diseases, with differing results. In LRIG1-positive non-small cell lung cancer, LMO7 was a negative prognostic marker [14], while another study of lung adenocarcinoma showed that the loss of LMO7 was associated with a worse prognosis [18]. It was also deemed tumor-promoting in pancreatic cancer [20]. In vulvar cancer, high LMO7 expression was associated with better overall survival (OS) and disease-free survival (DFS) [21]. This connection was stronger in non-HPV-driven tumors [21]. LMO7 has not been studied in OPSCC, and thus, the aim of this study was to elucidate the role of LMO7 in OPSCC and investigate its association with LRIG1 and other known prognostic clinicopathologic characteristics.

## Materials and methods

### Patients and specimens

Patients undergoing treatment for oropharyngeal cancer at Umeå University Hospital between 1990 and 2013 were identified via the Swedish Cancer Registry and patient records. Formalin-fixed, paraffin-embedded (FFPE) tumor tissue samples were retrieved from the biobank at Västerbotten County Council after approval from the Regional Ethical Review Board at Umeå University (Dnr 2013–475-31 M). Informed consent from participants alive at the start of the study was obtained. Clinical data were collected from patients’ records by a medical doctor. Out of initially 329 eligible patients treated with an intention to cure, 36 declined to participate, 34 were excluded due to insufficient clinical data and in 114 cases material could not be obtained. Thus, 145 patients were included in the study. Individual patients may be excluded from the respective molecular analyses due to missing data/lack of material.

### HPV DNA analysis

DNA was extracted from FFPE sections using DNA Tissue FFPE Tissue Kit or QIAamp MINI Kit (Qiagen, Inc., Valencia, CA, USA) according to the manufacturer’s instructions. DNA samples were stored at -20°C. HPV-PCR analysis was carried out using 100 ng of extracted DNA from each patient. General primers GP5 + /6 + were used to amplify a fragment of the conserved HPV L1 gene as previously described [22]. Amplification was performed in a Biometra Professional Thermocycler (Thermo Fisher Scientific, Waltham, MA, USA) or a T100 Thermal Cycler (Bio-Rad, Hercules, CA, USA). PCR products were run on a 2.5% agarose gel in a buffer consisting of 50 mM Tris/37 mM Borate/1.3 mM EDTA and stained with 0.5X GelRed (Biotium, Hayward, CA, USA). Gels were visualized under UV light. Fragments of 130‑150 bp were considered HPV-positive. To avoid false HPV-negative results due to disruption of the L1 gene when using the GP5 + /6 + primers, negative samples were reanalyzed with the general primers CpI/IIG as described [23]. Samples with products of approximately 188 bp were then considered positive.

HPV typing using HPV16 type-specific primers was also performed, and samples positive for HPV with general primers and negative for HPV16 were sequenced for determination of HPV type on the amplicon generated from these primers, as previously described [24].

### Immunohistochemistry

FFPE Sects. (4 µm) were deparaffinized, rehydrated, and rinsed in water. Immunohistochemistry (IHC) was performed using the Ventana standard procedure in a Ventana BenchMark ULTRA (Ventana Medical Systems, Tucson, AZ, USA). Antigen retrieval was performed with CC1 buffer. The following antibodies were used in the study: rabbit anti-LRIG1-Vina (product. no. AS184165; AgriSera AB, Vännäs, Sweden), 22 µg/ml; rabbit anti-LMO7 (HPA020923; Sigma, St. Louis, MO, USA), 2 µg/ml; mouse monoclonal anti-p16^INK4a^(E6H4) (Ventana Medical Systems), 1 µg/ml. Monoclonal mouse anti-Human CD44, phagocytic glycoprotein-1, clone DF1485, M7082 (Dako, Glostrup, Denmark) 2.6 µg/ml; monoclonal mouse anti-human Ki-67 antigen, clone MIB-1, M7240 (Dako), 1.6 µg/ml; Novocastra™ Liquid Mouse Monoclonal Antibody p53 Protein (DO-7), NCL-L-p53-DO7 (Leica Biosystems, Newcastle Upon Tyne, UK), 0.44 µg/ml. Validation of anti-LRIG1 and anti-LMO7 antibodies has been published previously [21]. Immunohistochemical slides were scanned on the Pannoramic Scan P250 FLASH III (3DHistech, Budapest, Hungary) with 40 × optical equivalent magnification.

### Evaluation and classification of immunostaining

LRIG1, LMO7, CD44, and p53 staining was scored based on both staining intensity and percentage of immunoreactive epithelial cells. The intensity was evaluated on a four-grade semiquantitative scale; ‘no staining’, ‘weak’, ‘intermediate’ or ‘strong intensity’, and the fraction of positive cells was scored separately and evaluated as 0%, 1–25%, 26–50%, 51–75%, and 76–100%. Ki67 staining was scored as the percentage of tumor cells with positive nuclear staining and the tumors were subgrouped into ‘at or above mean’ and ‘below mean’. The fraction and intensity of p16-positive cells were evaluated and only cases with strong and continuous nuclear and cytoplasmic expression in > 70% of positive cells were considered p16-positive [25]. Cases with cytoplasmic staining only were considered negative. Tumors with no or high (> 75%) P53 expression, or strong staining intensity, were considered p53 mutated in accordance with a previous study [26]. All evaluations were performed by an independent pathologist, with experience in interpreting LMO7 and LRIG staining, blinded to the clinical data and outcomes.

### Statistical analyses

DFS was defined from the date of completed treatment to the date of recurrence (according to pathological anatomical diagnosis or date of first recorded clinical progression). Death without documented recurrence was censored at the date of death. OS was defined as the time from the date of completed treatment to the date of death irrespective of the cause. If a patient was still alive at the time of accessing the patient’s records, the case was censored at that date.

Patient characteristics were analysed with the independent samples t-test or one-way ANOVA for comparison of means, or the Pearson chi-square test or Fisher’s exact test for ordinal variables. Two-sided *P* values were reported. DFS and OS were illustrated in Kaplan–Meier graphs and a log-rank test was used for comparison of the survival probabilities. Univariate Cox proportional hazard models were used to calculate the hazard ratio (HR). All variables with significant results in the univariate analysis were further tested in a Cox regression multivariable analysis to evaluate the independent factors' influence on the risk of death. To avoid overfitting, in some cases, variables with significant results in the univariate analysis were further investigated through backward stepwise elimination to estimate adjusted HRs. *P* values < 0.05 were considered statistically significant. All statistical analyses were performed using SPSS 28 software (IBM SPSS Statistics 28, IBM, Armonk, NY, USA).

## Results

### Study population

A total of 145 patients treated for oropharyngeal cancer with curative intent at Umeå University Hospital, Sweden, between 1990 and 2013 from whom clinical data and representative material could be obtained were enrolled in the study. The main characteristics of the study patients are summarized in Table 1. The patients were initially staged according to the 7th edition of the TNM staging classification and later restaged according to the Union for International Cancer Control (UICC) 8th edition [3]. p16 positivity causes downstaging of the tumor compared to the previous staging system, and this was also the case in our study (Table 1). The nodal status of the patients was as follows: N0 = 28, N1 = 11, N2 = 81, N2a = 2, N2b = 5, N2c = 6, N3 = 9, and N3b = 1. All patients were treated with radiotherapy (conventional or accelerated); in two cases the treatment was interrupted, and the intention was changed to palliation. Six patients underwent diagnostic surgery before radiotherapy, and 54 patients underwent surgery (neck dissection), the majority following radiotherapy. One patient had neoadjuvant chemotherapy, and nine patients were treated with concomitant chemotherapy. A total of 14/143 (10%) patients had residual tumor at the first follow-up, and 34/143 (24%) relapsed during the study period. There were 61 deaths from any cause, of which 47 patients died from OPSCC (Table 1).

**Table 1:** Correlations between HPV status and different clinicopathologic characteristics

Variable		Frequency (%)			
*N*		143			
		HPV-driven	Non-HPV-driven	All	*P* value
Sex	Male	86 (79)	25 (71)	111	0.312^b^
	Female	22 (21)	10 (29)	32	
Age (years)	Range	39–87	34–82	34–87	0.066^a^
	Mean	58.5	62	59.5	
Stage TNM 7	I	2 (2)	1 (3)	3	0.666^c^
	II	4 (4)	2 (6)	6	
	III	12 (11)	5 (14)	17	
	IV	90 (83)	27 (77)	117	
Stage TNM 8	I	10 (9)	1 (3)	11	< 0.001^c^*
	II	72 (67)	6 (17)	78	
	III	26 (24)	8 (23)	34	
	IV	0	20 (57)	20	
Localization	Tonsillar cancer	86 (79)	15 (43)	101	< 0.001^b^*
	Base of tongue cancer	17 (16)	14 (40)	31	
	Other oropharyngeal cancer	5 (5)	6 (17)	11	
Differentiation grade	Low	50 (48)	7 (20)	57	0.011^b^*
	Moderate	48 (46)	22 (65)	70	
	High	6 (6)	5 (15)	11	
	NK	4	1	5	
Smoking habit	Smoker	58 (59)	28 (85)	86	0.007^b^*
	Nonsmoker	40 (41)	5 (15)	45	
	NK	10	2	12	
Recurrence	Yes	19 (18)	15 (43)	34	< 0.001^b^*
	No	82 (76)	13 (37)	95	
	Residual tumor	7 (6)	7 (20)	14	
Vital status	Alive	74 (68)	8 (23)	82	< 0.001^b^*
	Dead	34 (32)	27 (77)	61	
Cause of death	Cancer	23 (72)	24 (89)	47	0.106^b^
	Other	9 (28)	3 (11)	12	
	NK	2	-	2	
LMO7 expression	Absent	1 (1.5)	1 (4)	2	0.732^c^
	1–25%	18 (25.5)	5 (20)	23	
	26–50%	16 (22.5)	7 (28)	23	
	51–75%	11 (15.5)	5 (20)	16	
	76–100%	25 (35)	7 (28)	32	
	NK	37	10		
LMO7 intensity	No	1 (2)	1 (5)	2	0.884^c^
	Weak	12 (21)	4 (20)	16	
	Intermediate	37 (65)	13 (65)	50	
	Strong	7 (12)	2 (10)	9	
	NK	51	15	66	
LRIG1 expression	Absent	1 (1.5)	0	1	1^c^
	1–25%	0	0	0	
	26–50%	2 (3)	0	2	
	51–75%	4 (6)	1 (4)	5	
	76–100%	61 (89.5)	25 (96)	86	
	NK	40	9	49	
LRGI1 intensity	No	1 (1.5)	0	1	0.755^c^
	Weak	4 (6)	0	4	
	Intermediate	54 (77)	21 (81)	75	
	Strong	11 (15.5)	5 (19)	16	
	NK	38	9	47	
CD44 expression	Absent	9 (13)	1 (4)	10	0.048^b^*
	1–25%	26 (37)	3 (12)	29	
	26–50%	9 (13)	4 (16)	13	
	51–75%	13 (18.5)	7 (28)	20	
	76–100%	13 (18.5)	10 (40)	23	
	NK	38	10	48	
CD44 intensity	No	9 (13)	1 (4)	10	0.024^b^*
	Weak	24 (34)	5 (20)	29	
	Intermediate	28 (40)	9 (36)	37	
	Strong	9 (13)	10 (40)	19	
	NK	38	10	48	
Ki67 expression	Mean	56	43	52.5	0.06^a^
p53 expression	Wild-type	70 (98.5)	8 (31)	78	< 0.001^b^*
	Mutated	1 (1.5)	18 (69)	19	
	NK	37	9	46	

*HPV* human papillomavirus, *NK* not known, *LRIG* leucine-rich repeats and immunoglobulin-like domain, *LMO7* LIM domain 7 protein

^a^Independent samples t-test

^b^Chi^2^-test

^c^Fisher’s exact test

^*^ = *P* < 0.05

### HPV and p16 analyses and patient characteristics

Seventy-seven percent (111/143) of the tumors were HPV-positive, 81% (117/143) were p16-positive and 76% (108/143) were positive for both HPV and p16. Two tumors were HPV-positive and p16-negative. Eight tumors were p16-positive but HPV-negative. In two cases, HPV data could not be retrieved, and in two cases, data on p16 were missing. In two cases one variable was positive, but the other was missing; thus, these cases could not be categorized as HPV-driven or not and excluded from further analyses. p16 is used as a surrogate marker for HPV infection and in the UICC-8 staging system for the classification of OPSCC [3]. The p16 analysis was thus used for restaging. However, in this study, tumors positive for both HPV and p16 were regarded as HPV-driven, in accordance with [27]. From the 111 HPV-positive tumors, genotyping was performed on 92. In 19 cases, sufficient material was lacking. HPV16 was the most frequent type, found in 84 cases (75%). HPV33 was found in four cases, and HPV18, HPV26, HPV35, and HPV45 were found in one case each. HPV-driven tumors were significantly associated with earlier stages, tonsillar origin, lower differentiation grade, and lower percentage of smokers compared to the patients with non-HPV-driven tumors, and all HPV-driven tumors except one exhibited p53 wild-type staining pattern (Table 1).

### HPV status and associations with recurrence and survival

**Fig. 1:**
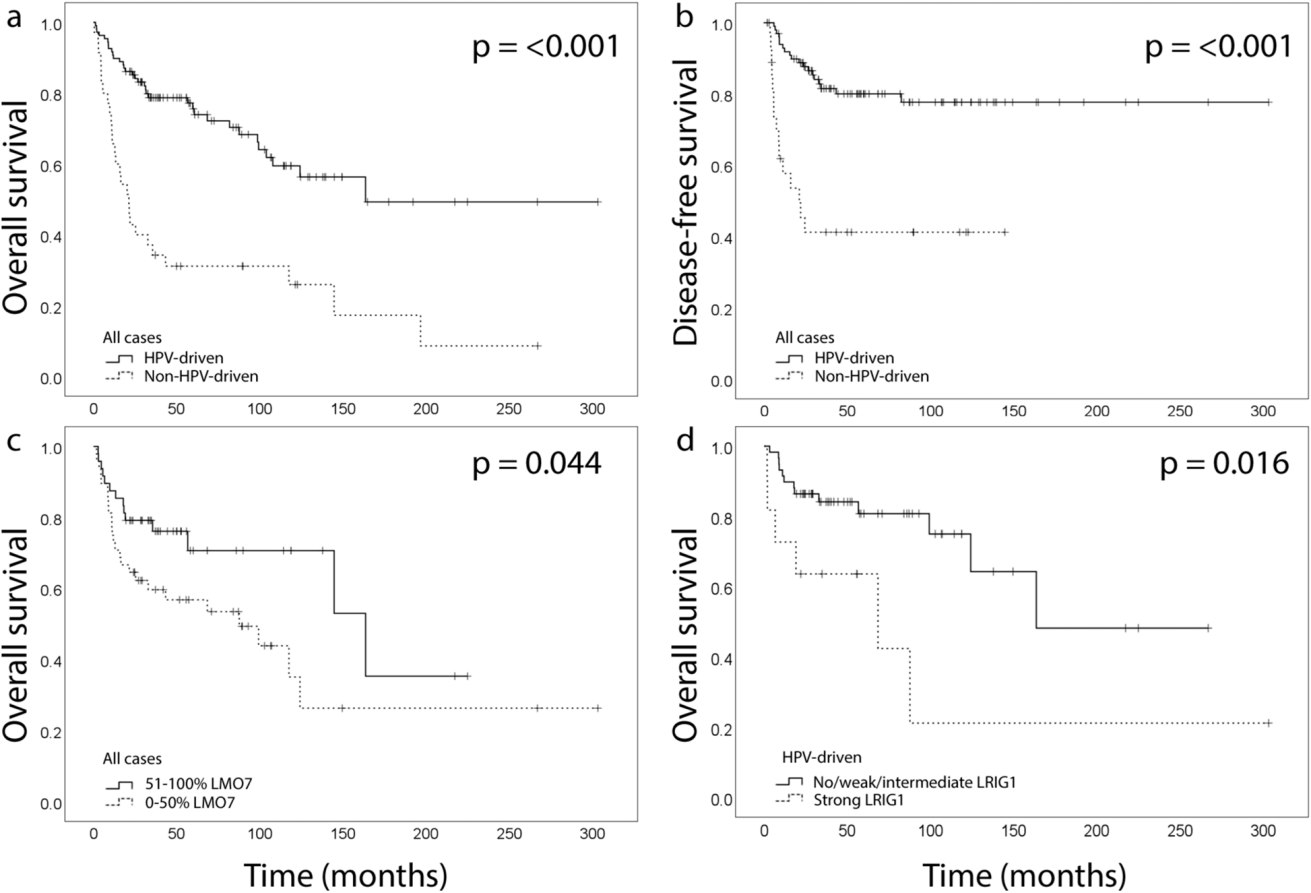
Kaplan–Meier curves showing overall survival (OS) and disease-free survival (DFS) according to HPV status, LMO7, and LRIG1 immunoreactivities, in the whole cohort and specified subgroups. **a** and **b** HPV-driven vs. non-HPV-driven tumors, OS and DFS, respectively, *n* = 143. **c** LMO7, all tumors, OS, *n* = 96. **d** LRIG1, HPV-driven tumors, OS, *n* = 70. HPV, human papillomavirus; LMO7, LIM domain 7 protein; LRIG, leucine-rich repeats and immunoglobulin-like domain

In the HPV-driven tumor group, 7 (6%) had residual tumor, 19 (18%) relapsed, and 34 (32%) died of any cause. In the group of non-HPV-driven tumors, 7 (20%) had residual disease, 15 (43%) relapsed and 27 (77%) died. HPV-driven tumors were associated with a significantly lower rate of recurrence (Table 1). HPV-driven tumors were associated with significantly longer OS and DFS (Fig. 1a and b).

### LMO7, LRIG1, CD44, Ki67 and p53 expression and their correlation to survival

**Fig. 2:**
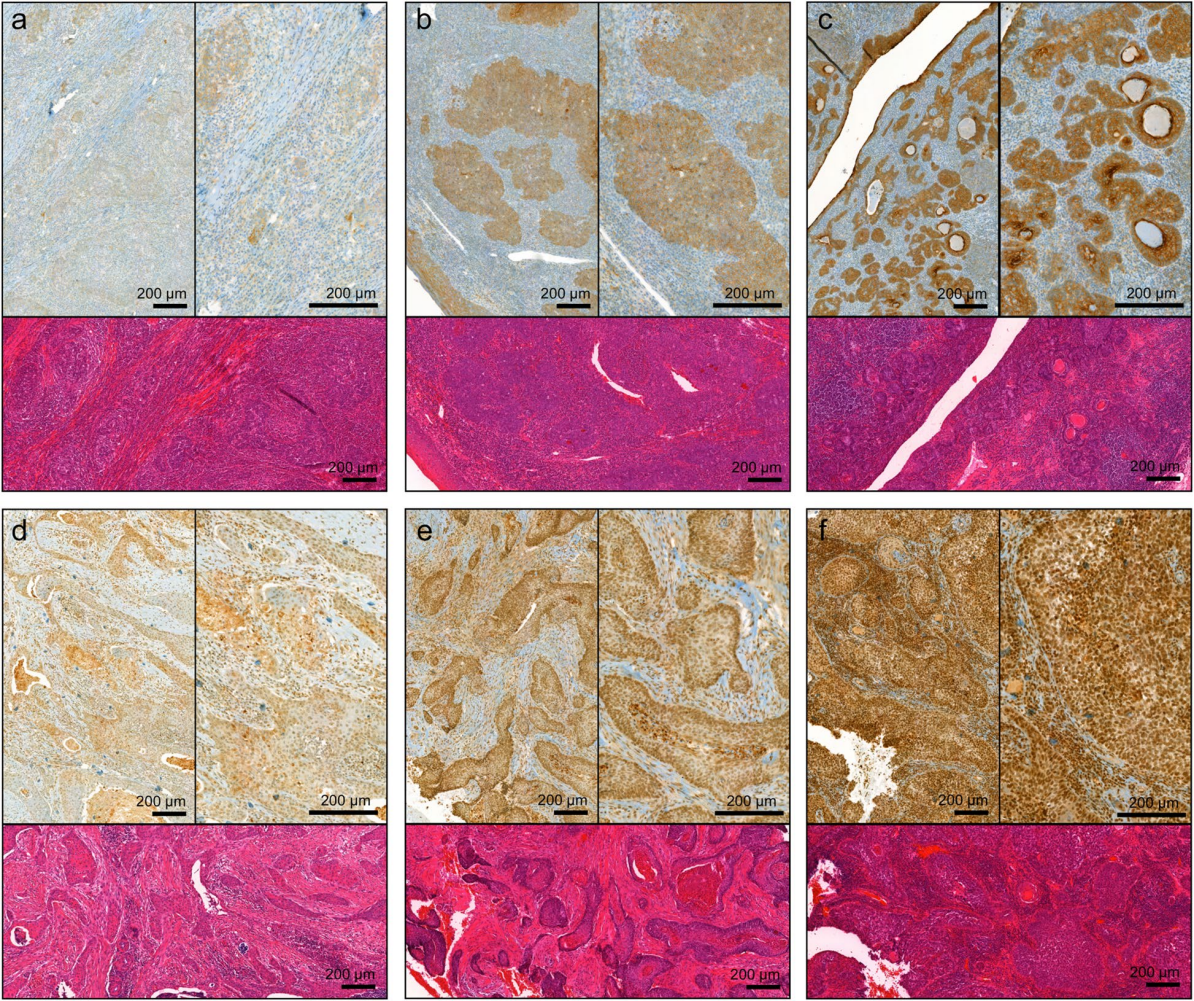
Microphotographs of representative tumor sections illustrating the hematoxylin and eosin staining and immunohistochemical staining of LMO7 and LRIG1. LMO7 staining intensity (brown) and percentage of positive cells; weak, 0–25% (**a**), intermediate, 26–75% (**b**), and strong, 76–100% (**c**). LRIG1 staining intensity (brown); weak (**d**), intermediate (**e**), and strong (**f**). Sections were counterstained with hematoxylin (blue). Scale bar 200 µm, 5 × and 10 × magnification. LMO7, LIM domain 7 protein; LRIG, leucine-rich repeats and immunoglobulin-like domain

**Fig. 3:**
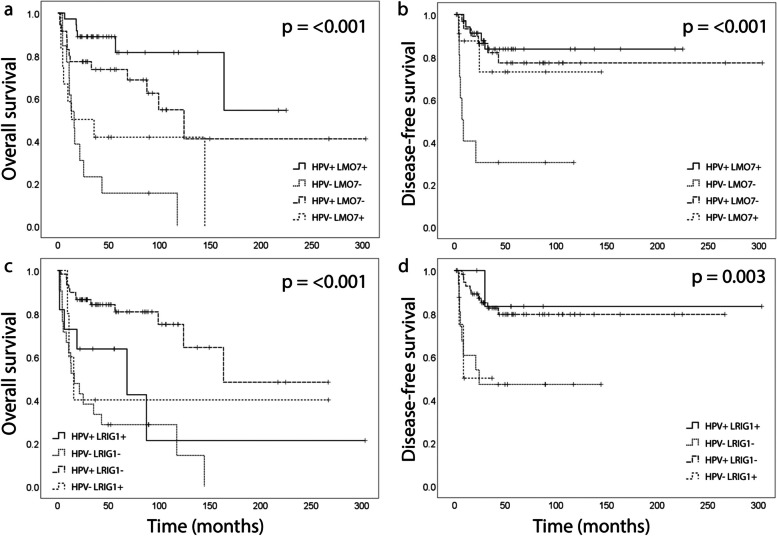
Kaplan–Meier curves showing overall survival (OS) and disease-free survival (DFS) in patients grouped according to the combined HPV and LMO7, or HPV and LRIG1 status, in the whole cohort. **a** and **b** comparing HPV-driven (HPV +) and LMO7 expression high (51–100%) (LMO7 +) tumors to non-HPV-driven (HPV-) and LMO7 expression low (0–50%) (LMO7-), and HPV-driven and LMO7 expression low, and non-HPV-driven and LMO7 expression high tumors, OS and DFS, respectively, *n* = 96. **c** and **d** comparing HPV-driven and LRIG1 staining intensity strong (LRIG1 +) tumors to non-HPV-driven and LRIG1 staining intensity no/weak/intermediate (LRIG1-), and HPV-driven and LRIG1 staining intensity strong, and non-HPV-driven and LRIG1 staining intensity no/weak/intermediate tumors, OS and DFS, respectively, *n* = 96

LMO7 staining intensity could be evaluated in 77/143 tumors and the number of positive cells in 96/143 tumors (exemplified in Fig. 2a-c). The staining intensity was weak in 16 (21%), intermediate in 50 (65%), and strong in 9 (12%) of the tumors; 23 (24%) of the tumors had 1–25% positive cells, 23 (24%) had 26–50%, 16 (17%) had 51–75% and 32 (33%) had 76–100% positive cells. There was no significant difference in the expression between HPV- and non-HPV-driven tumors (Table 1). When the staining was dichotomized as no/weak versus intermediate/strong, no/weak/intermediate versus strong, and 0–50% versus 51–100% no significant difference was found. LMO7 expression correlated with LRIG1 expression (*p* = 0.048), differentiation grade (*p* = 0.036) and vital status (*p* = 0.022) (Table 2). LMO7 expression did not correlate with any other molecular or clinical feature (Table 2). Survival analyses showed that a high (51–100%) staining percentage of LMO7 was associated with favorable OS (*p* = 0.044) (Fig. 1c). Stratification into HPV-/non-HPV-driven tumors did not reveal any difference within any of the groups. To further explore these results, we divided the patients into four groups according to their combined HPV and LMO7 status; HPV-driven tumors with high (51–100%) LMO7 expression (HPV + , LMO7 +), non-HPV-driven tumors with low (0–50%) LMO7 expression (HPV-, LMO7-), HPV-driven tumors with low LMO7 expression (HPV + , LMO7-) and non-HPV-driven tumors with high LMO7 expression (HPV-, LMO7 +). The clinical characteristics of the different groups can be viewed in Table 3. Survival analysis revealed that HPV + , LMO7 + tumors were associated with the best, and HPV-, LMO7- tumors with the worst, OS (Fig. 3a, *p* =  < 0.001). Patients with HPV-driven tumors that expressed a low percentage of LMO7 had the second-best survival and non-HPV-driven tumors with high LMO7 expression the third best. Analysis of DFS gave the same results (Fig. 3b, *p* =  < 0.001). There was no difference in OS or DFS between the tumors when grouped according to no/weak versus intermediate/strong staining intensity.

**Table 2:** Correlations between LMO7 expression and staining intensity and clinicopathologic characteristics

**Variable**		**LMO7 expression**		
		0–50% *n* (%)	51–100% *n* (%)	*P* value
Sex	Male	37 (47)	42 (53)	0.285^b^
	Female	11 (65)	6 (35)	
Stage TNM 8	I	1 (17)	5 (83)	0.177^c^
	II	28 (51)	27 (49)	
	III	14 (64)	8 (36)	
	IV	5 (38)	8 (62)	
Localization	Tonsil	33 (53)	29 (47)	0.492^c^
	Base of tongue	10 (40)	15 (60)	
	Other oropharyngeal	5 (56)	4 (44)	
Differentiation grade	Low	14 (37)	24 (63)	0.036^c^*
	Moderate	30 (62)	18 (38)	
	High	3 (33)	6 (67)	
	NK	1	0	
Smoking habit	Smoker	31 (56)	24 (44)	0.097^b^
	Nonsmoker	13 (38)	21 (62)	
	Unknown	4	3	
Recurrence	Yes	13 (65)	7 (35)	0.31^b^
	No	30 (45)	36 (55)	
	Residual tumor	5 (50)	5 (50)	
Vital status	Alive	23 (40)	34 (60)	0.022^b^*
	Dead	25 (64)	14 (36)	
Cause of death	Cancer	18 (62)	11 (38)	0.498^b^
	Other	6 (75)	2 (25)	
	Unknown	1	1	
LRIG1 expression	Absent	0	1 (100)	0.048^c^*
	1–25%	0	0	
	26–50%	1 (50)	1 (50)	
	51–75%	5 (100)	0	
	76–100%	40 (47)	45 (53)	
	NK	2	1	
LRIG1 intensity	No	0	1 (100)	0.744^c^
	Weak	3 (75)	1 (25)	
	Intermediate	37 (49)	38 (51)	
	Strong	8 (53)	7 (47)	
	NK	-	1	
LMO7 intensity	No	2 (100)	0	0.05^c^
	Weak	12 (75)	4 (25)	
	Intermediate	21 (42)	29 (58)	
	Strong	4 (44)	5 (56)	
	NK	9	10	
CD44 expression	Absent	7 (70)	3 (30)	0.275^b^
	1–25%	13 (45)	16 (55)	
	26–50%	4 (31)	9 (69)	
	51–75%	12 (63)	7 (37)	
	76–100%	11 (48)	12 (52)	
	NK	1	1	
CD44 intensity	No	7 (70)	3 (30)	0.347^b^
	Weak	13 (45)	16 (55)	
	Intermediate	16 (43)	21 (57)	
	Strong	11 (61)	7 (39)	
	NK	1	1	
Ki67 expression	$<$ Mean	33 (57)	25 (43)	0.093^b^
	$\ge$ Mean	14 (38)	23 (62)	
P53 expression	Wild-type	37 (48)	40 (52)	0.442^b^
	Mutated	11 (58)	8 (42)	

*LMO7* LIM domain 7 protein, *NK* not known, *LRIG* leucine-rich repeats and immunoglobulin-like domain

^a^Independent samples t-test

^b^Chi^2^-test

^c^Fisher’s exact test

^*^ = *P* < 0.05

**Table 3:** Correlations between HPV and LMO7 status and clinicopathologic characteristics

**Variable** *N*	**Frequency (%)** 96				
	HPV + , LMO7 +	HPV-, LMO7-	HPV + , LMO7 -	HPV -, LMO7 +	*P* value
Sex					
Male	31 (39)	9 (11.5)	28 (35.5)	11 (14)	0.454^a^
Female	5 (29)	4 (24)	7 (41)	1 (6)	
Age (years)					
Range	39–87	37–75	45–73	56–82	0.049^b^*
Mean	58.6	61.9	57.9	66.5	
Stage TNM 8					
I	4 (67)	0	1 (16.5)	1 (16.5)	< 0.001^a^*
II	26 (47)	3 (5.5)	25 (45.5)	1 (2)	
III	6 (27)	5 (23)	9 (41)	2 (9)	
IV	0	5 (38.5)	0	8 (61.5)	
Localization					
Tonsillar cancer	26 (42)	3 (5)	30 (48)	3 (5)	< 0.001^a^*
Base of tongue cancer	9 (36)	7 (28)	3 (12)	6 (24)	
Other oropharyngeal cancer	1 (11)	3 (33.5)	2 (22)	3 (33.5)	
Differentiation grade					
Low	21 (55)	1 (3)	13 (34)	3 (8)	0.005^a^*
Moderate	10 (21)	9 (19)	21 (44)	8 (16)	
High	5 (56)	2 (22)	1 (11)	1 (11)	
NK		1			
Smoking habit					
Smoker	15 (27)	11 (20)	20 (36.5)	9 (16.5)	0.029^a^*
Nonsmoker	19 (56)	2 (6)	11 (32)	2 (6)	
NK	2		4		
Recurrence					
Yes	5 (25)	7 (35)	6 (30)	2 (10)	0.017^a^*
No	29 (44)	4 (6)	26 (39)	7 (11)	
Residual tumor	2 (20)	2 (20)	3 (30)	3 (30)	
Vital status					
Alive	30 (52.5)	1 (2)	22 (38.5)	4 (7)	< 0.001^c^*
Dead	6 (15)	12 (31)	13 (33)	8 (21)

In the cohort of 143 patients, immunohistochemical LRIG1 expression was evaluated by staining intensity (exemplified in Fig. 2d-f) in 96 tumors and the fraction of positive cells in 94 tumors. LRIG1 staining was negative in 1 (1%) tumor, and the staining intensity was weak in 4 (4%), intermediate in 75 (78%), and strong in 16 (17%); 2 (2%) had 26–50% positive cells, 5 (5%) had 51–75% positive cells, and 86 (92%) had 76–100% positive cells. There was no significant difference in the expression between HPV- and non-HPV-driven tumors (Table 1). When the staining was dichotomized as absent/weak/intermediate versus strong or the percentage of positive cells as 0–75% versus 76–100% there was no significant difference in the expression between HPV- and non-HPV-driven tumors. There was no difference in OS or DFS when comparing tumors with a strong LRIG1 staining intensity to no/weak/intermediate. However, using HPV status as strata revealed a significantly worse OS in the patients with HPV-driven tumors when the tumors presented with a strong LRIG1 staining intensity (*p* = 0.016) (Fig. 1d). The patients were further grouped according to their combined HPV and LRIG1 status; HPV-driven tumors with strong LRIG1 staining intensity (HPV + , LRIG1 +), non-HPV-driven tumors with no/weak/intermediate LRIG1 staining intensity (HPV-, LRIG1-), HPV-driven tumors with no/weak/intermediate LRIG1 staining intensity (HPV + , LRIG1-) and non-HPV-driven tumors with strong LRIG1 staining intensity (HPV-, LRIG +). The clinical characteristics of the different groups can be seen in Table 4. Survival analysis showed that the HPV-driven, LRIG1 staining intensity no/weak/intermediate tumors were associated with the best OS (Fig. 3c, *p* =  < 0.001). The group with HPV-driven tumors with strong LRIG1 staining intensity had a better OS than the two groups of non-HPV-driven tumors. Studying DFS, the HPV-driven tumors had a beneficial prognosis, regardless of LRIG1 status (Fig. 3d, *p* = 0.003). When the percentage of positive cells was dichotomized as 0–75% versus 76–100% there was no significant difference in OS or DFS. No further difference was seen within the group of HPV- or non-HPV-driven tumors.

**Table 4:** Correlations between HPV and LRIG1 status and clinicopathologic characteristics

**Variable** *N*	**Frequency (%)** 96				
	HPV + , LIRG1 +	HPV-, LRIG1-	HPV + , LRIG1 -	HPV -, LRIG1 +	*P* value
Sex					
Male	9 (11)	18 (22)	50 (63)	3 (4)	0.516^a^
Female	2 (12)	4 (23)	9 (53)	2 (12)	
Age (years)					
Range	45–87	37–82	39–76	34–75	0.205^b^
Mean	59.4	63.8	58.2	59.6	
Stage TNM 8					
I	0	1 (17)	5 (83)	0	< 0.001^a^*
II	8 (15)	4 (7)	42 (76)	1 (2)	
III	3 (13.5)	5 (23)	12 (54.5)	2 (9)	
IV	0	11 (85)	0	2 (15)	
Localization					
Tonsillar cancer	6 (10)	3 (5)	50 (79)	4 (6)	< 0.001^a^*
Base of tongue cancer	3 (13)	12 (50)	8 (33)	1 (4)	
Other oropharyngeal cancer	2 (22)	6 (67)	1 (11)	0	
Differentiation grade					
Low	6 (16)	4 (11)	27 (73)	0	0.104^a^
Moderate	4 (8)	14 (29)	27 (55)	4 (8)	
High	1 (11)	2 (22)	5 (56)	1 (11)	
NA		1			
Smoking habit					
Smoker	6 (11)	17 (30)	29 (52)	4 (7)	0.032^a^*
Nonsmoker	2 (6)	4 (12)	27 (82)	0	
NK	3		3	1	
Recurrence					
Yes	1 (5)	8 (38)	10 (48)	2 (9)	0.019^a^*
No	8 (12)	9 (14)	46 (71)	2 (3)	
Residual tumor	2 (20)	4 (40)	3 (30)	1 (10)	
Vital status					
Alive	5 (9)	4 (7)	46 (80.5)	2 (3.5)	< 0.001^a^*
Dead	6 (15)	17 (44)	13 (33)	3 (8)	

*HPV* human papillomavirus, *LRIG* leucine-rich repeats and immunoglobulin-like domain, *HPV* + HPV-driven; *HPV-* non-HPV-driven, *LRIG1* + strong LRIG1 staining intensity, *LRIG1-* no/weak/intermediate LRIG1 staining intensity, *NK* not known

^a^Fisher’s exact test

^b^One-way ANOVA

^*^ = *P* < 0.05

**Fig. 4:**
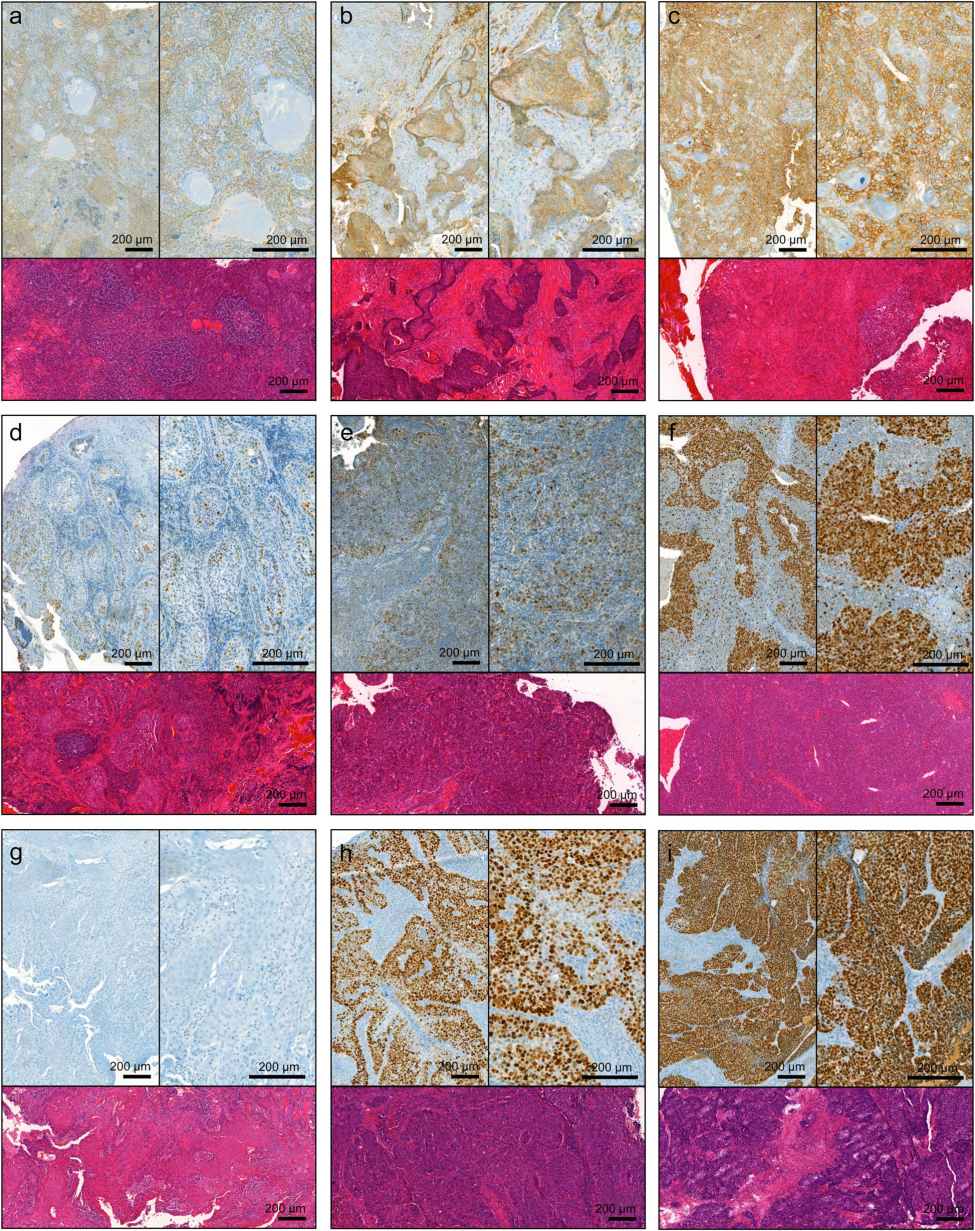
Microphotographs of representative tumor sections illustrating the hematoxylin and eosin staining and immunohistochemical staining of CD44, Ki67, and p53. CD44 staining intensity (brown) and percentage of positive cells; weak, 1–25% (**a**), intermediate, 26–50% (**b**), and strong, 51–75% (**c**). Ki67 percentage of positive cells (brown); 20% (**d**), 35% (**e**), and 95% (**f**). p53 staining intensity (brown); no staining (**g**), intermediate, 51–75% (**h**), and strong, 76–100% (**i**). Sections were counterstained with hematoxylin (blue). Scale bar 200 µm, 5 × and 10 × magnification

**Fig. 5:**
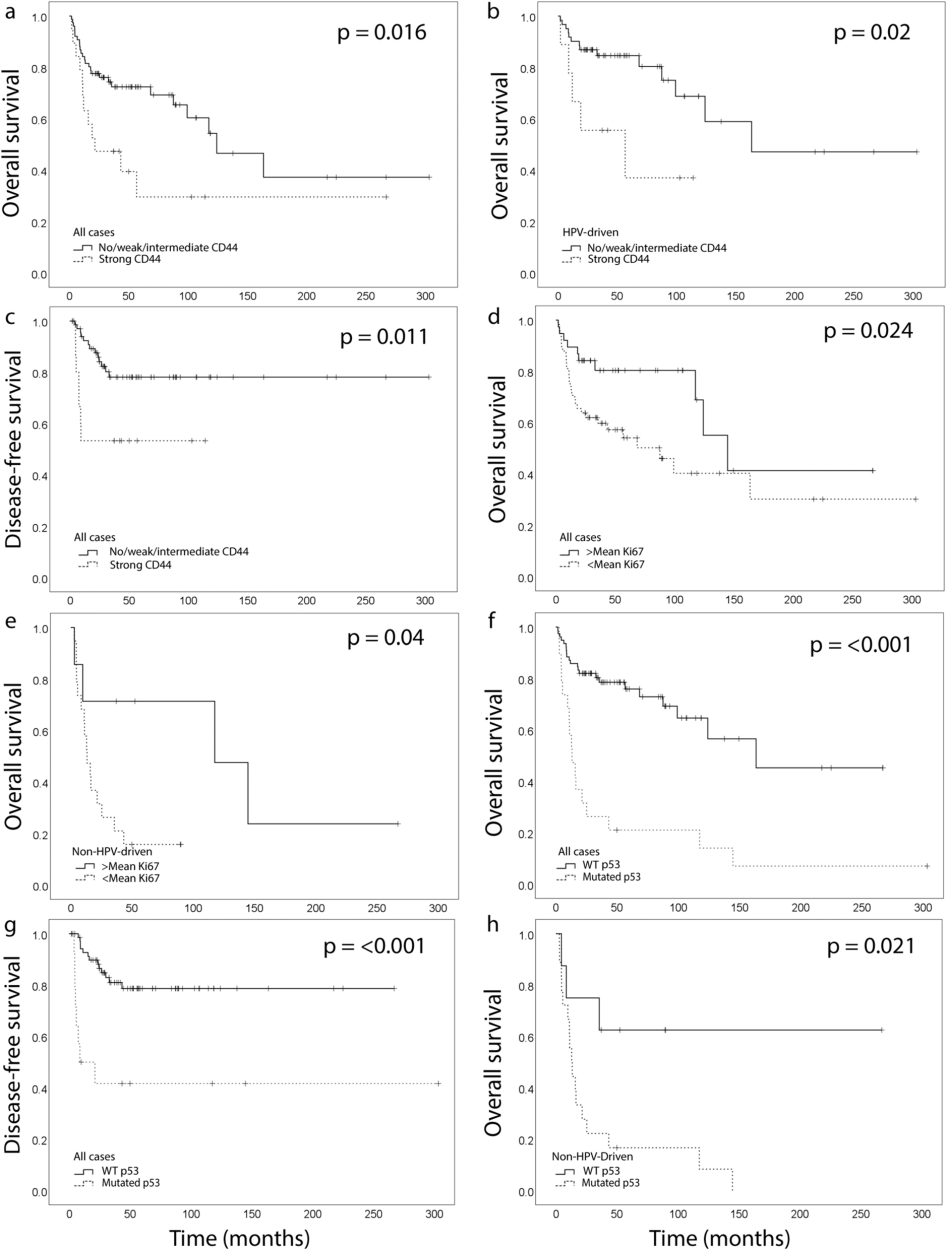
Kaplan–Meier curves showing overall survival (OS) and disease-free survival (DFS) according to CD44, Ki67, and p53 immunoreactivities, in the whole cohort and specified subgroups. **a** and (**b**) CD44, all tumors and HPV-driven tumors, respectively, OS, *n* = 95, and (**c**) all tumors, DFS. **d** Ki67, all tumors, OS, *n* = 96, and (**e**) non-HPV-driven tumors, OS, *n* = 26. **f** and (**g**) p53, all tumors, OS, and DFS, respectively, *n* = 97. **h** P53, non-HPV-driven tumors, OS, *n* = 26. WT, wild-type

Ninety-five tumor tissue samples were successfully stained for CD44 and evaluated both by staining intensity and fraction of positive cells (as exemplified in Fig. 4a-c) (Table 1). When the intensity was dichotomized as absent/weak versus medium/strong, the HPV-driven tumors more often expressed a lower staining intensity (33/70, 47% vs. 6/26, 24%, *p* = 0.043). When the fraction of positive cells was dichotomized as 0–25% versus > 25%, the HPV-driven tumors also expressed CD44 to a significantly lower extent (35/70, 50% vs. 21/25, 84%, *p* = 0.003). There was a significant correlation between strong CD44 staining intensity and poor overall survival (Fig. 5a, *p* = 0.016), which was also observed when analyzing the HPV-driven group of tumors separately (Fig. 5b, *p* = 0.02). Patients with tumors exhibiting a strong CD44 staining intensity also had a significantly shorter DFS (Fig. 5c, *p* = 0.011). There was no difference when CD44 intensity was dichotomized as no/weak versus intermediate/strong or the percentage of positive cells as 0–25% versus 26–100% or 0–50% versus 51–100%. Strata according to HPV status did not reveal further significant results regarding OS or DFS.

The expression of Ki67 was evaluated in 96/143 tumors (exemplified in Fig. 4d-f). The mean fraction of cells expressing Ki67 was 52.5%. In the HPV-driven tumors the mean was 56% (*n* = 70) and in the non-HPV-driven tumors the mean was 43% (*n* = 26). The HPV-driven tumors expressed Ki67 to a significantly higher extent than the non-HPV-driven tumors (Table 1). Using the mean as a cutoff, a high expression of Ki67-positive cells correlated with longer OS (Fig. 5d, *p* = 0.024). Stratifying patients according to HPV status gave the same results in the patient group of non-HPV-driven tumors (Fig. 5e, *p* = 0.04). No significant results were observed when analyzing DFS.

P53 expression intensity and the number of positive cells were evaluated in 97/143 tumors (exemplified in Fig. 4g-i). There was a significant difference between HPV-driven and non-HPV-driven tumors. All HPV-driven tumors except one (1.5%), exhibited p53 wild-type pattern, in contrast to the non-HPV-driven tumors where 18 (69%) were p53 mutated (Table 1). Survival analyses showed that p53 wild-type expression correlated with significantly longer OS (Fig. 5f, *p* =  < 0.001) and DFS (Fig. 5g, *p* =  < 0.001), in contrast to mutated p53 expression. Using HPV status as strata revealed that p53 wild-type expression was associated with longer OS (Fig. 5h, *p* = 0.021) also in the subgroup of non-HPV-driven tumors. No survival analysis was performed in the group of HPV-driven tumors since there was only one case of p53 mutation.

### Univariate analyses

The crude hazard ratio for overall survival is shown in Table 5. There was no association between OS and sex, smoking, or LRIG1 staining intensity. There was a significant association between age, tumor localization, differentiation grade, stage according to TNM 8, HPV status, LMO7 expression, CD44 staining intensity, Ki67 and p53 expression, and OS. Patients with a high percentage (51–100%) of LMO7-positive cells had a favorable OS (HR 0.515, 95% CI: 0.267–0.994, *p* = 0.048). The univariate associations of patient and disease characteristics with disease-free survival are shown in Table 6. Stage according to TNM 8, HPV status, CD44 staining intensity, and p53 expression correlated with DFS. Grouping the patients according to HPV status revealed that in the HPV-driven tumors (108/143) (Table 7), there was an association between OS and age, stage according to TNM 8, differentiation grade, LRIG1, and CD44 staining intensity. There were no significant associations between DFS and the different patient and disease characteristics (data not shown). In the non-HPV-driven tumors (35/143), univariate analyses showed that p53 mutated tumors were associated with shorter OS (HR 3.881, 95% Cl: 1.124–13.404, *p* = 0.032, data not shown). There were no other significant association with DFS or OS (data not shown).

**Table 5:** Cox regression univariate and multivariate associations of clinicopathologic characteristics with overall survival

Overall survival						
	Univariate analysis			Multivariate analysis		
Variable	HR	95% CI	*P* value	aHR	95% CI	*P* value
*Sex*						
Male	1	(ref)				
Female	0.88	0.482–1.606	0.677			
*Age*						
< mean	1	(ref)		1	(ref)	
> mean	2.456	1.455–4.144	< 0.001*	2.314	1.094–4.898	0.028*
*Smoking habit*						
Nonsmoker	1	(ref)				
Smoker	1.855	0.998–3.447	0.051			
*Localization*						
Tonsil	1	(ref)				
Base of tongue	1.367	0.74–2.526	0.318			
Other	4.071	1.943–8.531	< 0.001*			
*Differentiation grade*						
Low	1	(ref)				
Moderate	2.485	1.365–4.525	0.003*			
High	1.785	0.642–4.962	0.266			
*Stage TNM 8*						
I-II	1	(ref)		1	(ref)	
III-IV	4.217	2.47–7.199	< 0.001*	2.915	1.221–6.961	0.016*
*HPV status*						
HPV-driven	1	(ref)		1	(ref)	
Not HPV-driven	3.463	2.083–5.759	< 0.001*	1.771	0.777–4.036	0.174
*LMO7 expression*						
Low (0–50%)	1	(ref)		1	(ref)	
High (51–100%)	0.515	0.267–0.994	0.048*	0.586	0.297–1.153	0.122
*LRIG1 staining intensity*						
No/weak/intermediate	1	(ref)				
Strong	1.644	0.777–3.478	0.194			
*CD44 staining intensity*						
No/weak/intermediate	1	(ref)				
Strong	2.272	1.144–4.515	0.019*			
*Ki67 expression*						
$<$ mean	1	(ref)		1	(ref)	
$\ge$ mean	0.446	0.217–0.918	0.028*	0.418	0.193–0.906	0.027*
*p53 expression*						
Wild-type	1	(ref)				
Mutated	4.476	2.358–8.497	< 0.001*			

*HPV* human papilloma virus, *LMO7* LIM domain 7 protein, *LRIG* leucine-rich repeats and immunoglobulin-like domain

^*^ = *P* < 0.05

**Table 6:** Cox regression univariate and multivariate associations of clinicopathologic characteristics with disease-free survival

Disease-free survival						
	Univariate analysis			Multivariate analysis		
Variable	HR	95% CI	*P* value	aHR	95% CI	*P* value
*Sex*						
Male	1	(ref)				
Female	0.915	0.398–2.102	0.834			
*Age*						
< mean	1	(ref)				
> mean	1.147	0.581–2.261	0.693			
*Smoking habit*						
Nonsmoker	1	(ref)				
Smoker	2.275	0.984–5.261	0.055			
*Localization*						
Tonsil	1	(ref)				
Base of tongue	1.175	0.506–2.728	0.707			
Other	2.324	0.699–7.725	0.169			
*Differentiation grade*						
Low	1	(ref)				
Moderate	2.121	0.998–4.508	0.051			
High	1.356	0.297–6.197	0.694			
*Stage TNM 8*						
I-II	1	(ref)		1	(ref)	
III-IV	3.525	1.78–6.983	< 0.001*	1.717	0.595–4.958	0.317
*HPV status*						
HPV-driven	1	(ref)		1	(ref)	
Not HPV-driven	4.529	2.295–8.94	< 0.001*	1.806	0.428–7.615	0.421
*LMO7 expression*						
Low (0–50%)	1	(ref)				
High (51–100%)	0.468	0.187–1.175	0.106			
*LRIG1 staining intensity*						
No/weak/intermediate	1	(ref)				
Strong	1.139	0.336–3.87	0.834			
*CD44 staining intensity*						
no/weak/intermediate	1	(ref)		1	(ref)	
strong	3.126	1.243–7.858	< 0.015*	1.596	0.591–4.308	0.356
*Ki67 expression*						
$<$ mean	1	(ref)				
$\ge$ mean	0.642	0.259–1.591	0.338			
*p53 expression*						
Wild-type	1	(ref)		1	(ref)	
Mutated	5.118	2.109–12.424	< 0.001*	2.849	0.757–10.727	0.122

*HPV* human papillomavirus, *LMO7* LIM domain 7 protein, *LRIG* leucine-rich repeats and immunoglobulin-like domain

^*^ = *P* < 0.05

**Table 7:** Cox regression univariate and multivariate associations of overall survival with clinicopathologic characteristics, in HPV-driven tumors

HPV-driven tumors, overall survival						
	Univariate analysis			Multivariate analysis		
Variable	HR	95% CI	*P*-value	aHR	95% CI	*P*-value
*Sex*						
Male	1	(ref)				
Female	0.816	0.337–1.975	0.652			
*Age*						
< mean	1	(ref)		1	(ref)	
> mean	2.958	1.459–5.999	0.003*	2.93	1.072–8.005	0.036*
*Smoking habit*						
Nonsmoker	1	(ref)				
Smoker	1.343	0.634–2.842	0.441			
*Localization*						
Tonsil	1	(ref)				
Base of tongue	0.347	0.083–1.455	0.148			
Other	2.853	0.862–9.444	0.086			
*Differentiation grade*						
Low	1	(ref)				
Moderate	1.775	0.843–3.738	0.131			
High	0.571	0.073–4.475	0.594			
*Stage TNM 8*						
I-II	1	(ref)		1	(ref)	
III-IV	3.123	1.569–6.217	0.001*	3.871	1.474–10.167	0.006*
*LMO7 expression*						
Low (0–50%)	1	(ref)				
High (51–100%)	0.438	0.166–1.160	0.097			
*LRIG1 staining intensity*						
No/weak/intermediate	1	(ref)		1	(ref)	
Strong	3.114	1.178–8.232	0.022*	2.847	1.036–7.825	0.043*
*CD44 staining intensity*						
no/weak/intermediate	1	(ref)				
strong	3.22	1.132–9.154	0.028*			
*Ki67 expression*						
$<$ mean	1	(ref)				
$\ge$ mean	0.605	0.229–1.6	0.311			

*HPV* human papilloma virus, *LRIG* leucine-rich repeats and immunoglobulin-like domain, *LMO7* LIM domain 7 protein

^*^ = *P* < 0.05

### Multivariate analysis

Parameters with a significant correlation to OS or DFS, respectively, in the univariate analyses were included in the multivariate analysis. To avoid overfitting regarding overall survival, backward stepwise selection was used to select which of the variables to include in the final multivariate analysis (Tables 5 and 7). This analysis revealed that stage according to the 8th edition of the UICC TNM staging system and Ki67 expression were the only independent prognostic indicators of OS. There were no independent prognostic factors for DFS (Table 6). In the group of patients with HPV-driven tumors (Table 7), the multivariate analysis revealed that age, stage according to TNM 8, and LRIG1 staining intensity were independent prognostic factors of OS. Strong LRIG1 staining intensity was associated with a worse prognosis (HR 2.847, 95% Cl: 1.036–7.825, *p* = 0.043).

## Discussion

In this report, we evaluated the prognostic impact of LMO7 in relation to previously known prognostic markers through immunohistochemical staining of FFPE material and the collection of clinical data from 145 patients diagnosed with OPSCC. To our knowledge, this is the first report investigating the role of LMO7 in OPSCC. We show that high LMO7 expression was associated with significantly better OS (*p* = 0.044). Combining HPV and LMO7 status in survival analyses revealed four groups with significantly different OS (Fig. 3a). The beneficial effect of a high LMO7 expression may in part explain the difference in prognosis seen within the group of patients with HPV-driven tumor. When grouping was performed by combining HPV and LRIG1 status the difference was not as pronounced. We thus suggest that LMO7 primarily needs to be further explored as a potential biomarker for risk-stratifying patients within the HPV-driven and non-HPV-driven OPSCC tumor groups respectively. LMO7 was a positive prognostic factor for OS in OPSCC in univariate analysis (HR 0.515, 95% CI: 0.267–0.994, *p* = 0.048). LMO7 expression also correlated with LRIG1 expression (*p* = 0.048), consistent with previous findings [14]. Interestingly, LRIG1 was an independent negative prognostic factor in the HPV-driven group of tumors (HR 2.847, 95% Cl: 1.036–7.825, *p* = 0.043). No conclusions on LMO7 expression could be drawn when stratifying for HPV status. In the group of non-HPV-driven tumors, p53 expression was the only significant prognostic factor. However, because of the limited number of cases (*n* = 35) in the analyzed cohort, the possible role of LMO7 as a prognostic marker in this group cannot be ruled out. The connection between LMO7 and HPV has to our knowledge previously only been studied in vulvar cancer [21]. The authors found that a high LMO7 expression was associated with a favorable OS in the group of HPV-negative patients (*n* = 70). Considering this and our results from the present study, the connection between HPV and LMO7 should be further investigated in a larger cohort of OPSCC patients.

LRIG1 is generally considered a positive prognostic factor, as seen in vaginal carcinoma [12], prostate [28], breast [29, 30], lung [31], and uterine cervical cancer [13, 32], among others [7]. LRIG1 is also a positive prognostic marker in OPSCC; Lindquist et al*.* (2014) found that LRIG1 was associated with longer OS and DFS in OPSCC in the whole cohort and in the subgroup of HPV-driven tumors [11]. In contrast, our results indicate that LRIG1 staining intensity is a negative prognostic factor in HPV-driven tumors. The LRIG1 antibody used in this study has previously been used in an investigation of LRIG1 expression in vulvar cancer. In line with our results, this study showed that no/weak LRIG1 staining in HPV-negative tumors was associated with a better OS [21]. The discrepancy between these two reports and previous findings might be due to methodological reasons. Both the LRIG1-Vina antibodies used in this cohort and the LRIG1-151 antibodies used in the study of Lindquist et al. were polyclonal, targeting the same antigen, and the same procedure was used in both studies. Thus, we can justify the result as follows: evaluation of staining intensity is highly subjective, and some factors influence the staining, such as the duration of storage of the tumor section, fixation method of the tumor tissue, and methodology of the immunohistochemistry. Our results indicate that there is a need for more standardized antibody development and interpretation of the result, which needs to be tested in a larger material before conclusions can be drawn on LRIG1 in OPSCC to be used in a clinical setting. Nonetheless, high LRIG1 expression is not consistently favorable and it has been suggested that its function can be either tumor-promoting or suppressive depending on the cellular context [33]. In an untreated prostate cancer cohort, LRIG1 was a marker for poor survival [28]. In contrast, LRIG1 was a positive prognostic factor following prostatectomy, further indicating that the association between survival and LRIG1 is context-dependent [28]. Blood levels of LRIG1 have only recently been studied, and interestingly, the results from a high-grade serous ovarian cancer cohort showed that high levels were associated with poor prognosis [34]. The authors speculate that this could be connected to the BMP-promoting effect of LRIG1, as BMPs are associated with tumor-stimulating functions in ovarian carcinoma [34]. Neither the role of BMPs nor the association between BMPs and LRIG1 has been studied in OPSCC. Overall, the conflicting results on the role of LRIG1 in OPSCC warrant further studies.

As expected, HPV-driven tumors were associated with longer DFS and OS (*p* =  < 0.001). The current staging system stratifies patients according to HPV status based on p16 expression via IHC, which is used as a surrogate marker [35]. However, approximately 20% of p16-positive tumors may lack transcriptionally active HPV, which may affect outcome [36]. It has also been proposed that transient HPV infection may cause cases where HPV DNA can be detected but p16 is negative [37]. Studies indicate that a positive assessment of p16 (via IHC) should be confirmed by high-risk HPV DNA detection [36], and this is recommended in the current Swedish guidelines [38]. Thus, we chose to consider p16-positive/HPV-positive tumors as HPV-driven.

CD44 is a transmembrane glycoprotein overexpressed in several tumors. Binding of its main ligand hyaluronan causes proliferation, survival and motility-enhancing signaling [39]. Absent/weak CD44 staining intensity has been found to correlate with a longer OS and DFS in OPSCC [40, 41], and this was also confirmed in our material (Fig. 5a-c), as was the finding that HPV-driven tumors express both a lower CD44 staining intensity and a lower percentage of positive cells than non-HPV-driven tumors (Table 1) [40]. The HPV-driven tumors expressed Ki67 to a significantly higher extent than the non-HPV-driven tumors (Table 1), consistent with previous studies [42]. Surprisingly, our data suggest that Ki67 expression at or above the mean is associated with favorable prognosis, also in multivariate analysis (Table 5), in contrast to other studies [42, 43]. There is no consensus on what cutoff to use which makes it difficult to compare the results and thus this needs to be further investigated. Different studies use different cutoffs regarding p53 staining intensity and expression [44, 45]. Two reports on HPV-associated gynecological cancers suggest that tumors with no p53 staining, strong staining intensity, or a high expression of positive cells should be considered p53 mutated [46, 47]. This division has also been used in a recent report on oropharyngeal carcinoma [26]. In concordance with this study [26], all HPV-driven tumors, except one, presented with p53 wild-type expression (Table 1). Survival curves illustrated worse OS and DFS in tumors with mutated p53 expression (Fig. 5f-g), and univariate Cox regression revealed a significant association between mutated p53 expression and worse OS (Table 5) and DFS (Table 6), as seen before [44].

## Conclusions

LMO7 immunoreactivity was found for the first time to be a positive predictor of OS in OPSCC, and its role as a biomarker should be further investigated. In the group of HPV-driven tumors, strong LRIG1 staining intensity was associated with worse OS and it was shown to be a negative prognostic factor in univariate and multivariate analysis, in contrast to a previous report [11]. Further studies are needed on the mechanisms and clinical applicability of LMO7 and LRIG proteins in OPSCC.

## Data Availability

The datasets used and/or analyzed during the current study are available from the corresponding author on reasonable request.

## Abbreviations

OPSCC: Oropharyngeal squamous cell carcinoma
HPV: Human papillomavirus
TNM: Tumor, node, metastasis
LRIG: Leucine-rich repeats and immunoglobulin-like domains
LMO7: LIM domain 7 protein
OS: Overall survival
DFS: Disease-free survival
FFPE: Formalin-fixed, paraffin-embedded
IHC: Immunohistochemistry
UICC: Union for International Cancer Control
HR: Hazard ratio
WT: Wild-type
